# Receipt of preventive health services and current cannabis users

**DOI:** 10.1080/10550887.2021.1967688

**Published:** 2021-08-25

**Authors:** Emily Lum, Sri Lekha Tummalapalli, Meena Khare, Salomeh Keyhani

**Affiliations:** a Northern California Institute for Research and Education, San Francisco, CA, USA;; b Division of Healthcare Delivery Science & Innovation, Department of Population Health Sciences, Weill Cornell Medicine, New York, NY, USA;; c Department of Medicine, University of California, San Francisco, CA, USA;; d San Francisco Veterans Administration, San Francisco, CA, USA

**Keywords:** Cannabis, preventive health services, Marijuana

## Abstract

Substance use is associated with greater barriers and reduced access to care. Little research, however, has examined the relationship between cannabis use and receipt of preventive health services. Using data from the 2017 Behavioral Risk Factor Surveillance System, we examined the association between current cannabis use and receipt of 12 preventive health services, adjusting for sociodemographic characteristics and access to care. In analyses that adjusted for sociodemographic factors and access to care, participants with current cannabis use had lower odds of being vaccinated for influenza (AOR = 0.67, 95% CI = 0.54–0.83) and higher odds of ever receiving HPV vaccination (AOR = 1.77, 95% CI = 1.06–2.96) and HIV screening (AOR = 2.34, 95% CI = 1.88–2.92) compared with those without cannabis use. Among the 12 preventive services examined, we found three differences in receipt of preventive services by cannabis use status. Cannabis use does not appear to be associated with significant underuse of preventive services.

## Introduction

Cannabis is legal for some form of use in 33 states and Washington, D.C. Legalization has been accompanied by increased cannabis use. Between 2001–2002 and 2012–2013, past year cannabis use doubled from 4.1% to 9.5% in U.S. adults.^1^ A 2017 study found that approximately 15% and 8% of U.S. adults had used cannabis in the past year and past 30 days, respectively.^2^

Prior research has demonstrated that heavy use of alcohol and/or drugs is associated with greater barriers and reduced access to health care.^3^ No studies to date have examined the association of cannabis use with access to care. Cannabis has adverse neurocognitive effects, including decreased functional connectivity, activity, and volume in regions of the brain associated with learning, memory, and inhibitory control.^4^ Persistent cannabis use is associated with memory problems, difficulty managing activities of daily living, and amotivation.^5,6^ Cannabis use also has mental health effects, including an association with psychosis, treatment relapse for depression, and avoidance of social situations in those with social anxiety.^4,7^

The social, neurocognitive, and mental health effects of cannabis may be associated with less engagement with health care services. Little research, however, has examined the relationship between cannabis use and health promoting behaviors such as obtaining preventive care. Therefore, in this study, we examined the association of current cannabis use with receipt of preventive health services.

## Methods

We used data from the 2017 Behavioral Risk Factor Surveillance System (BRFSS). We included states that participated in both the optional Marijuana Module and other optional modules on cardiovascular health, vaccinations, alcohol screening, and diabetes. Our predictor was current cannabis use, as assessed by the question “During the past 30 days, on how many days did you use marijuana or hashish?” We examined uptake of 12 preventive services, including cardiovascular risk reduction strategies, vaccinations, health behavior screenings, and diabetes care (Table 2). We used recommended guidelines from the U.S. Preventive Services Task Force (USPSTF), the Centers for Disease Control and Prevention (CDC), or the American Diabetes Association (ADA) to determine eligible participants (Table 2). Using chi-squared statistics and logistic regression, we examined whether participants who had used cannabis in the past 30 days were more or less likely than those who did not use cannabis to receive preventive health services. We adjusted for sociodemographic characteristics, including age, sex, race/ethnicity, employment, education, marital status, and access to care (insurance status and having a personal doctor).

The 2017 BRFSS data are publicly available and exempt from Institutional Review Board approval.

## Results

Overall, 56,924 individuals (unweighted) were included, representing 45,655,241 U.S. adults. Of those, 10.7% reported current cannabis use. Participants with cannabis use were more likely to be younger, male, unmarried, and non-Hispanic white and had fewer comorbidities compared to those who did not use cannabis (Table 1). Additionally, those with current cannabis use were less likely than those who did not use cannabis to have health insurance (84.9% vs. 88.9%, *p* = 0.002) or a personal doctor (64.7% vs. 78.5%, *p* < 0.001).

In analyses that adjusted for sociodemographic factors and access to care, participants with current cannabis use had lower odds of being vaccinated for influenza in the past year (adjusted odds ratio [AOR] = 0.67, 95% confidence interval [CI] = 0.54–0.83) and higher odds of ever receiving HPV vaccination (AOR = 1.77, 95% CI = 1.06–2.96) and HIV screening (AOR = 2.34, 95% CI = 1.88–2.92) compared with those without cannabis use. There were no statistically significant differences in receipt of serum cholesterol, alcohol use, or diabetes screenings between participants with and without current cannabis use in adjusted analyses.

## Discussion and conclusions

We examined the relationship between current cannabis use and receipt of preventive services. Among the 12 services examined, participants with current cannabis use were less likely to receive influenza vaccination but more likely to receive HPV vaccination and HIV screening than those who did not use cannabis. Prior analyses have shown that those who use cannabis are more likely to engage in unprotected sex.^8^ This difference in health behaviors may explain why they were more likely to receive HPV vaccination and HIV screening. We also found that participants with cannabis use were less likely to have insurance coverage suggesting that access to care may also be a factor in the association between cannabis use and receipt of preventive care services.

Some limitations are noted. This study was limited to individuals in 10 states and Washington, D.C. who participated in the Marijuana Module and other select optional modules in 2017, thus potentially limiting the overall generalizability of our findings. However, the states that were included are geographically diverse. Receipt of preventive services are also by self-report. Cannabis use information was limited to the past 30 days, so we were unable to assess the relationship between cumulative cannabis use and receipt of preventive health services. Additionally, we included participants in our analysis who reported any cannabis use in the past 30 days, which may explain why we found little association between cannabis use and receipt of preventive services. An analysis limited to daily use in the past 30 days may demonstrate a different relationship. Finally, we did not account for the use of other substances in the analysis. However, this is an important relationship to examine in future work as people with more frequent cannabis use are more likely to use other substances^9^ and use of other substances is associated with lower likelihood of receiving preventive health services.^10^

In conclusion, among the 12 preventive measures identified, we found three differences in receipt of preventive health services by cannabis use status. Current cannabis use does not appear to be associated with significant underuse of preventive services.

## Figures and Tables

**Table 1.** Comparison of characteristics of participants with and without current cannabis use.

Variable	Unweighted *n* = 56924	Weighted *N* = 45655241	Current cannabis use (%)	No cannabis use (%)	*p*-Value
**Age**					
18–29	5266	8948243	39.6	17.2	<0.001
30–49	13223	15737392	38.0	34.1	
50–64	17732	11710029	15.9	26.8	
≥65	20703	9259577	6.6	21.9	
**Sex**					
Male	25129	21929777	61.6	46.4	<0.001
**Race/ethnicity**					
Non-Hispanic White	44535	25196515	58.3	55.8	<0.001
Non-Hispanic Black	5114	4753569	12.9	10.3	
Hispanic	3008	9475614	18.2	21.4	
Asian, Native Hawaiian, or Pacific Islander	827	4050649	5.3	9.5	
Other	2359	1433773	5.2	2.9	
**Employed**	28186	25793136	63.8	56.0	<0.001
**Education level**					
Less than high school graduate	4099	6748417	9.6	15.5	<0.001
High school graduate	15115	11488459	28.8	24.8	
College and post-doc training	37518	27246866	61.6	59.7	
**Married**	30406	23232570	27.9	54.0	<0.001
**Clinical characteristics and medical history**					
Obesity (BMI ≥30)	16773	12467698	22.8	30.0	<0.001
Hypertension^a^	23285	14489481	23.3	32.8	<0.001
Hyperlipidemia^b^	20499	13488084	21.7	33.3	<0.001
Diabetes^c^	7722	5227443	4.5	12.3	<0.001
Cardiovascular Disease (CVD)^d,e^	6527	3571282	5.0	8.2	0.004
**Combined above five chronic conditions**					
None	19312	19489603	56.1	41.1	<0.001
1 condition	15510	12191209	24.1	27.0	
2 conditions	11419	7399397	11.6	16.8	
3–5 conditions	10678	6573769	8.2	15.1	
**Insurance**					
Health coverage^f^	51944	40227767	84.9	88.9	0.002
Has a personal doctor^g^	46866	35024660	64.7	78.5	<0.001

a Have you EVER been told by a doctor, nurse, or other health professional that you have high blood pressure?.

b Have you EVER been told by a doctor, nurse, or other health professional that your blood cholesterol is high?.

c (Ever told) you have diabetes?.

d (Ever told) you that you had a heart attack also called a myocardial infarction?; (Ever told) you had angina or coronary heart disease?; (Ever told) you had a stroke?.

e Includes coronary artery disease or angina, stroke, and myocardial infarction.

f Do you have any kind of health care coverage, including health insurance, prepaid plans such as HMOs, government plans such as Medicare, or Indian Health Service?.

g Do you have one person you think of as your personal doctor or health care provider?.

**Table 2.** Comparison of receipt of preventive health measures in participants with and without current cannabis use.

Preventive health service	Numerator	Denominator	Current cannabis use (unweighted *n* = 3169, weighted *N* = 4880798) (%)	No cannabis use (unweighted *n* = 53755, weighted *N* = 40774443) (%)	*p*-Value	Unadjusted OR (95% CI)	Adjusted OR^a^ (95% CI)
**Cardiovascular risk reduction**							
Serum cholesterol screening^b^	Cholesterol checked in past 5 years	Participants age 40–75	88.7	93.7	0.001	0.53 (0.36, 0.78)	0.81 (0.54, 1.21)
Serum cholesterol checked^b^	Cholesterol checked in past 5 years	Participants age 40–75 years with hx of HTN, HLD, or CVD	85.3	91.0	0.006	0.57 (0.38, 0.86)	0.84 (0.57, 1.24)
Aspirin use^b,c^	Regular aspirin use	Participants age 50–69 years excluding if aspirin is unsafe	39.5	40.2	0.91	0.97 (0.57, 1.65)	1.07 (0.60, 1.91)
**Vaccination**							
Influenza vaccination	Influenza vaccination in past year	All participants	25.4	41.5	<0.001	0.48 (0.40, 0.59)	0.67 (0.54, 0.83)
Pneumonia vaccination	Pneumonia vaccination ever	Participants age ≥65	70.2	72.5	0.69	0.90 (0.52, 1.55)	0.95 (0.55, 1.65)
Tetanus vaccine	Tetanus vaccination since 2005	All participants	32.6	36.6	0.26	0.84 (0.62, 1.14)	0.78 (0.56, 1.1)
HPV vaccination	HPV vaccination ever	Participants age 18–26	40.5	36.6	0.47	1.18 (0.75, 1.85)	1.77 (1.06, 2.96)
**Other health screening**							
Blood sugar checked^b^	Test for high blood sugar or diabetes in past 3 years	Participants age 40–70 years who are overweight or obese	41.5	58.0	<0.001	0.51 (0.44, 0.60)	0.88 (0.73, 1.05)
Alcohol use screening^b^	Asked if you drink alcohol during checkup	All participants	87.4	78.0	0.001	1.96 (1.30, 2.97)	1.56 (0.99, 2.46)
HIV test or screening^d^	Ever tested for HIV	Participants age 18–65	63.2	43.2	<0.001	2.26 (1.86, 2.75)	2.34 (1.88, 2.92)
**Diabetes care (among participants with diabetes only)**							
Serum cholesterol checked^e^	Cholesterol checked in past 5 years	History of DM	93.5	95.8	0.40	0.63 (0.21, 1.86)	1.97 (0.41, 9.55)
HbA1c checked^e^	HbA1c measured in past year	History of DM	27.1	12.0	0.042	2.73 (0.99, 7.53)	2.22 (0.97, 5.09)
Foot exam^a^	Foot exam in past year	History of DM	33.7	33.3	0.97	1.02 (0.44, 2.32)	1.62 (0.68, 3.88)
Eye exam^a^	Dilated eye exam in past year	History of DM	48.8	57.8	0.37	0.70 (0.32, 1.54)	1.25 (0.59, 2.64)
Influenza vaccination^c^	Influenza vaccination in past year	History of DM	35.9	56.9	0.014	0.42 (0.21, 0.87)	0.58 (0.27, 1.24)
Pneumonia vaccination^c^	Pneumonia vaccination ever	History of DM	58.5	58.2	0.97	1.02 (0.45, 2.30)	1.96 (0.88, 4.35)

a Adjusted for age, sex, race/ethnicity, employment, education, marital status, insurance status, and having a personal doctor.

b “The USPSTF recommends the service. There is high certainty that the net benefit is moderate or there is moderate certainty that the net benefit is moderate to substantial” or “Supportive evidence from well-conducted cohort studies or Supportive evidence from a well-conducted case-control study” (ADA).

c “The USPSTF recommends selectively offering or providing this service to individual patients based on professional judgment and patient preferences. There is at least moderate certainty that the net benefit is small” or “Supportive evidence from poorly controlled or uncontrolled studies or Conflicting evidence with the weight of evidence supporting the recommendation” (ADA).

d “The USPSTF recommends the service. There is high certainty that the net benefit is substantial” or “Clear evidence from well-conducted, generalizable, randomized controlled trials that are adequately powered or Supportive evidence from well-conducted randomized controlled trials that are adequately powered or Compelling nonexperimental evidence” (ADA).

e “Expert consensus or clinical experience” (ADA).

ADA, American Diabetes Association; BRFSS, Behavioral Risk Factor Surveillance System; CVD, cardiovascular disease; DM, diabetes mellitus; HbA1C, hemoglobin A1c; HIV, human immunodeficiency virus; HPV, human papilloma virus; HLD, hyperlipidemia; HTN, hypertension; USPSTF, United States Preventative Services Task Force.
